# *PDE11A* gene polymorphism in testicular cancer: sperm parameters and hormonal profile

**DOI:** 10.1007/s40618-021-01534-3

**Published:** 2021-03-04

**Authors:** F. Faja, F. Finocchi, T. Carlini, F. Rizzo, F. Pallotti, M. Spaziani, G. Balercia, A. Lenzi, D. Paoli, F. Lombardo

**Affiliations:** 1 Laboratory of Seminology - “Loredana Gandini” Sperm Bank, Department of Experimental Medicine, “Sapienza” University of Rome, Viale del Policlinico 155, 00161 Rome, Italy; 2 Division of Endocrinology, Department of Clinical and Molecular Sciences, Umberto I Hospital, Polytechnic University of Marche, Ancona, Italy; 3 Hormone Laboratory, Department of Experimental Medicine - Medical Pathophysiology Section, “Sapienza” University of Rome, Rome, Italy

**Keywords:** Testicular germ cell tumours, Spermatogenesis, Hormones, *PDE11A*, Polymorphisms, cAMP signaling

## Abstract

**Purpose:**

Testicular germ cell tumours (TGCTs) is the most common malignancy among young adult males. The etiology is multifactorial and both environmental and genetic factors play an important role in the origin and development of TGCT. Genetic susceptibility may result from the interaction of multiple common and low-penetrance genetic variants and one of the main candidate genes is *PDE11A*. Many *PDE11A* polymorphisms were found responsible for a reduced PDE activity in TGCT patients, who often also display impaired hormone and sperm profile. The aim of this study was to investigate testicular function and *PDE11A* sequence in testicular cancer cases.

**Methods:**

Semen analysis was performed in 116 patients with unilateral and bilateral sporadic TGCTs and in 120 cancer-free controls. We also investigated hormone profile and *PDE11A* polymorphisms using peripheral blood samples.

**Results:**

Our data revealed that TGCT patients showed lower testosterone levels, higher gonadotropins levels and worse semen quality than controls, although the mean and the medians of sperm parameters are within the reference limits. *PDE11A* sequencing detected ten polymorphisms not yet associated with TGCTs before. Among these, G223A in homozygosity and A288G in heterozygosity were significantly associated with a lower risk of testicular tumour and they displayed a positive correlation with total sperm number.

**Conclusions:**

Our findings highlight the key role of *PDE11A* in testis and suggest the presence of an underlying complex and fine molecular mechanism which controls testis-specific gene expression and susceptibility to testicular cancer.

## Introduction

Testicular germ cell tumours (TGCTs) represent the most common solid malignancy in men of reproductive age with an initial peak in childhood and a second, much larger peak beginning immediately after puberty [1]. They comprise about 95% of testicular tumours and their incidence has increased 3–4 times over the last 50 years. TGCTs are classified into two categories based on the presence of one or more histological types: tumours with a single histological type (seminomas and non-seminomas) that represent about 40% of all testicular neoplasms and tumours displaying two or more histological types (mixed tumours) that are the remaining 60%.

Histopathological studies have shown that most TGCTs arise from germ cell neoplasia in situ [2]. Dynamic epigenetic changes occur during normal development of germ cells and expression of genes which regulate this process is tightly controlled by epigenomic mechanisms, such as DNA methylation and microRNA [3–7]. Therefore, any genetic and environmental factor disturbing the maturation of primordial germ cells (PGCs) or gonocytes could induce the onset of TGCTs.

TGCTs can affect one (unilateral) or both testes (bilateral) simultaneously (synchronous forms) or after a certain period of time from the first testicular manifestation (metachronous forms) [8]. The presence of a unilateral testicular tumour is one of the most important risk factor for the development of a malignant cancer in the contralateral organ [9]. As bilateral testicular tumours are often diagnosed in young men with a familiarity for TGCTs, a genetic component can be present at the base of these neoplasms. However, the etiology is multifactorial and, in addition to the genetic contribution, risk factors, such as exposure to endocrine disruptors (EDs) and cryptorchidism, could play a key role in carcinogenesis. In particular, EDs could cause the development of TGCTs by disturbing the synthesis, release, transport, metabolism, binding, action or elimination of endogenous hormones during embryonic development [10, 11].

Although the molecular causes of these tumours remain elusive, evidences in literature support the hypothesis of a genetic contribution for the development of TGCTs with *PDE11A* as a possible candidate gene [12–14]. This gene encodes a dual-specificity phosphodiesterase (PDE) capable of hydrolyzing both cAMP and cGMP and isoform 4 is highly expressed in testicular tissue, which so far is the only known tissue expressing all four *PDE11A* isoforms [15]. *Pde11a* knockout mice display male infertility, a factor associated to increased TGCT risk [16–19]. Moreover, alterations in the cAMP pathway have also been observed in non-germ cell-derived testicular tumours [20, 21], suggesting the importance of this pathway in testicular tissue. Interestingly, two recent studies investigated the putative link between testicular tumours and *PDE11A* polymorphisms (SNPs), finding that inactivating *PDE11A* variants seem to be associated with TGCT risk in both familial and sporadic cases [12, 14].

In the light of the aforementioned evidences, the aim of our study was:

- to evaluate hormone profile and sperm parameters to assess testicular function in 116 patients with unilateral and bilateral sporadic TGCTs compared to 120 cancer-free controls;
- to investigate *PDE11A* SNPs in our caseload to confirm the association between testicular tumours and two missense variants identified in previous studies, p.V820M and p.K568R.

## Materials and methods

### Patients

The study was approved by our University Hospital’s Institutional Review Board (Ethical Committee of “Sapienza” University of Rome—Azienda Ospedaliera Policlinico Umberto I) and all patients gave their informed written consent.

We enrolled 116 Caucasian patients with unilateral or bilateral sporadic TGCTs (Group T) attending the Laboratory of Seminology—Sperm Bank “Loredana Gandini”, Department of Experimental Medicine at “Sapienza” University of Rome for semen cryopreservation. As controls, we recruited 120 cancer-free Caucasian men (Group C), attending our laboratory for semen analysis as part of an andrological work-up for preconceptional screening. Men with a history of azoospermia, hypogonadism and known genetic diseases were excluded from the study. For the two groups, we estimated both sperm parameters and serum hormone levels. Moreover, we performed molecular analysis to identify *PDE11A* SNPs.

### Semen analysis

Semen samples were collected by masturbation after 3–5 days of abstinence. All samples were allowed to liquefy at 37 °C for 60 min and were then assessed according to WHO 2010 [22]. The following variables were taken into consideration: ejaculate volume (ml), sperm concentration (10^6^ per ml), total sperm number (10^6^ per ejaculate), progressive motility (%) and morphology (% abnormal forms).

### Hormone profile

A peripheral blood sample was collected from each subject at 8 a.m. after overnight fasting to measure serum levels of follicle-stimulating hormone (FSH), luteinizing hormone (LH) and testosterone. Hormones were quantified by Chemiluminescent Microparticle ImmunoAssay (CMIA, Architect System; Abbott Laboratories, Abbott Park, IL, USA), with detection limits of 0.05 UI/L, 0.07 UI/L, 0.28 nmol/L for FSH, LH and testosterone, respectively. Intra and inter-assay coefficients of variation were 3.1% and 7.0% at 3.2 UI/L (FSH), 3.6% and 5.1% at 3.3 UI/L (LH), 2.1% and 3.6% at 10.08 nmol/L (testosterone), respectively. In our laboratory, normal ranges for adults were 1.38–9.58 UI/L (FSH), 1.80–8.16 UI/L (LH) and 9.4–33.5 nmol/L (testosterone), respectively.

### *PDE11A* sequencing

Blood samples underwent following steps to identify *PDE11A* SNPs. Firstly, DNA was extracted from peripheral blood leukocytes using Wizard Genomic DNA Purification Kit (Promega, Madison, WI, USA). Extracted DNA was quantified by NanoDrop ND-2000 (Thermo Fisher Scientific, Waltham, MA, USA) and underwent molecular analysis to perform *PDE11A* sequencing based on Sanger method.

We identified a region of *PDE11A* in which the SNPs rs140269105 (p.V820M) and rs148955609 (p.K568R), previously identified in literature [12, 14], are present. Primer pairs were designed about 200 bp upstream and downstream of the 2 SNPs of interest using the software primer 3 plus. We refer to Fragment1 for the region containing the SNP p.V820M and Fragment2 for the region containing the SNP p.K568R. The primer sequences were as follows:

- Fragment1_F 5′-GGGCTGTGCAATAAACTGTG-3’
- Fragment1_R 5′-ATAAACAGTGCTGCCCCTTG-3’
- Fragment2_F 5′-GAATGGGCTTCAAGGCATCT-3’
- Fragment2_R 5′-ATGTGCCTATTTCCCCAAGG-3’

The amplification reaction was carried out using 30 ng of genomic DNA in 50 µl under the following PCR conditions: 10 min at 95 °C followed by 35 cycles of 1 min at 94 °C, 1 min at 58 °C, 1 min and 20 s at 72 °C and a final extension step at 72 °C for 12 min. A 5 µl of each PCR product was then used for electrophoresis on 2% agarose gel to check the presence and exact length of the amplified fragments (389 and 444 nucleotides for Fragment1 and Fragment2, respectively).

The fragments were then purified using PureLink PCR Purification Kit (Invitrogen, Life Tecnologies, USA) and the sequencing was performed with Big Dye Terminator® v1.1 Cycle Sequencing Kit (Applied Biosystems, Foster City, CA, USA). To separate the end-labeling reaction products from the unused dye-terminators, salts and other low molecular weight products, Centri-Sep® gel filtration columns (Applied Biosystems, USA) were used. Finally, the labelled fragments underwent capillary electrophoresis using the 3500 Genetic Analyzer sequencer (Applied Biosystems).

### Bioinformatic analysis

Raw data from the capillary electrophoresis were analysed by Sequencing Analysis v5.1 (Applied Biosystem, USA). To improve reliability and exclude the presence of artefacts, each sample was sequenced twice using the same primers in the two directions 5′–3′ and 3′–5′. To detect *PDE11A* SNPs, samples sequences were compared against the reference sequence GRCh38.p12 (NG_012168.2) in GenBank using sequence alignment (Blast). All SNPs discovered were compared with the lists in dbSNP database (https://www.ncbi.nlm.nih.gov/snp). The impact of all SNPs detected on coding sequence was studied to understand if they were synonymous or not. Furthermore, we verified if they were already identified in the previous literature and if they were associated with pathological conditions. The software used was Geneious v.R 9.0.2.

### Statistical analysis

Continuous variables are presented as mean ± SD or as median and interquartile range, as appropriate, after evaluation of the normality of distributions using the Kolmogorov–Smirnov test. Student’s T or Mann–Whitney U test have been used to compare sperm parameters between the two groups. Categorical variables are presented as counts and/or percentages and differences in frequencies are performed by the χ^2^ test. The presence of statistically significant correlations among sperm parameters and hormone levels was evaluated using Spearman’s rank correlation test.

Binary logistic regression models have been used to analyze associations between testicular cancer and *PDE11A* SNPs. Finally, associations between sperm parameters, hormone levels and polymorphisms have been investigated by univariate generalized linear models.

*PDE11A* alleles frequencies were determined by the gene counting method and the agreement of the genotype distribution with the Hardy–Weinberg Equilibrium test was calculated. Differences of the SNPs frequencies between the study groups have been evaluated by the χ^2^ test. A two-tailed *P value* ≤ 0.05 was considered significant. All computations were carried out with Statistical Package for the Social Sciences (SPSS) 25.0 (SPSS Inc., Chicago, USA).

## Results

### Study population and histological data

We studied 116 patients affected by unilateral or bilateral sporadic TGCTs (Group T) and 120 cancer-free controls (Group C), aged 32.6 ± 7.3 and 27.8 ± 7.2 years, respectively (*P* < 0.001). No significant differences between Group T and Group C were found in Body Mass Index (BMI) (24.7 ± 3.8 vs. 24.2 ± 3.2 kg/m^2^ respectively, *P* = 0.355) and percentage of smokers (25.0% vs. 21.7%, respectively, *P* = 0.647).

Group T was composed by:

- 5 patients with synchronous bilateral neoplasm;
- 13 patients with contralateral metachronous recurrence neoplasm;
- 98 patients with unilateral neoplasm (of which 65% was seminomas and the remaining 35% was mixed tumours and non-seminomas).

In particular, metachronous neoplasms appeared after a median of 4 years and these patients had been treated with 2–4 cycles of cisplatin, bleomycin and etoposide (PEB) in three cases, a single cycle of radiotherapy in other three cases and only follow up in the seven remaining cases.

### Semen analysis

Comparison of the sperm parameters of the two study groups revealed a poor semen quality in Group T (Table 1). Except for the ejaculate volume (3.1 ± 1.6 vs. 2.9 ± 1.1 ml in Group T and Group C, respectively, *P* = 0.699), Group T showed a significantly lower total sperm number (93.5 ± 99.2 vs. 178.0 ± 171.5 × 10^6^/ejaculate in Group T and Group C, respectively, *P* < 0.001), progressive motility (34.1 ± 19.3% vs. 42.3 ± 18.1%, in Group T and Group C, respectively, *P* < 0.001) and a higher percentage of abnormal forms (89.6 ± 13.3% vs. 87.7 ± 7.2% in Group T and Group C, respectively, *P* < 0.001). In particular, in Group T 40% of samples appears oligozoospermic in contrast to 23.5% of samples in Group C.

**Table 1:** Mean ± SD, median (in brackets), 25th to 75th percentile distribution in italics, significance of the sperm parameters (Mann–Whitney U test) and percentage of oligozoospermic patients (*χ*^2^ test) in the two study groups

	Semen volume (mL)	Sperm concentration (10^6^/mL)	Total sperm number (10^6^/ejaculate)	Progressive motility (%)	Abnormal forms (%)	Oligozoospermic (%)
Group T	3.1 ± 1.6 (3.0) *2.0–4.0*	30.5 ± 27.7 (25.0) *8.0–46.5*	93.5 ± 99.2 (54.3) *19.0–153.0*	34.1 ± 19.3 (40.0) *17.5–50.0*	89.6 ± 13.3 (90.0) *88.0–95.5*	40.0
Group C	2.9 ± 1.1 (3.0) *2.0–3.5*	65.0 ± 58.9 (52.0) *15.0–95.0*	178.0 ± 171.5 (140.0) *45.0–256.0*	42.3 ± 18.1 (50.0) *30.0–55.0*	87.7 ± 7.2 (88.0) *82.0–93.0*	23.5
*P* value	0.699	** < 0.001**	** < 0.001**	** < 0.001**	** < 0.001**	**0.008**

*Group T*, patients with testicular germ cell tumours; *Group C*, cancer-free controls

Significant *P* values are in bold

It is worth stressing that in both groups the means and the medians of sperm parameters are within the reference limits according to WHO 2010.

Furthermore, in the caseload as a whole, progressive motility and abnormal forms are weakly correlated with age (progressive motility:* ρ* = − 0.184, *P* = 0.004; abnormal forms: * ρ* = 0.220, *P* = 0.001) and, except for the morphology, with BMI (total sperm number: * ρ* = −0.153, *P* = 0.026; progressive motility: * ρ* = −0.144, *P* = 0.036).

### Hormone parameters

The hormone dosage revealed a significant worse profile in Group T (Table 2). In particular, testosterone level was significantly lower (17.7 ± 6.4 vs. 21.6 ± 6.5 nmol/ml in Group T and Group C, respectively, *P* < 0.001), while the levels of gonadotropins were significantly higher (FSH: 10.4 ± 7.2 vs. 4.1 ± 3.6 mUI/ml, *P* < 0.001; LH: 5.5 ± 4.2 vs. 3.8 ± 1.7 mUI/ml, *P* < 0.001, in Group T and Group C, respectively).

**Table 2:** Mean ± SD, median (in brackets), 25th to 75th percentile distribution in italics and significance of the hormone levels in the two study groups (Mann–Whitney U test)

	FSH (mUI/ml)	LH (mUI/ml)	Testosterone (nmol/ml)
Group T	10.4 ± 7.2 (8.4) *6.2*–*12.3*	5.5 ± 4.2 (4.5) *3.3*–*6.7*	17.7 ± 6.4 (17.0) *13.3*–*21.4*
Group C	4.1 ± 3.6 (3.1) *2.2*–*5.2*	3.8 ± 1.7 (3.4) *2.6*–*4.8*	21.6 ± 6.5 (20.7) *17.7*–*25.5*
*P* value	** < 0.001**	** < 0.001**	** < 0.001**

*Group T*, patients with testicular germ cell tumours; *Group C*, cancer-free controls; *FSH*, follicle-stimulating hormone; *LH*, luteinizing hormone

Significant *P* values are in bold

As expected, hormone levels were significantly correlated with both age (FSH: * ρ* = 0.439, *P* < 0.001; LH: * ρ* = 0.240, *P* = 0.001; testosterone: * ρ* =  − 0.186, *P* = 0.010) and BMI (FSH: * ρ* = 0.180, *P* = 0.019; LH: * ρ* = 0.245, *P* = 0.001; testosterone: * ρ* = −0.260, *P* = 0.001).

### Genetic study of *PDE11A*

*PDE11A* sequencing did not reveal the two missense variants investigated (p.V820M and p.K568R) in the caseload. However, we detected the following ten polymorphisms:

- For the Fragment1: C207T, G223A, A288G, T366C;
- For the Fragment2: C102A, G172A, C189T, T245C, C255A, G371C.

Sequencing analysis of Fragment1 was carried out in 116 patients with TGCTs and in 120 subjects from Group C. Instead, sequencing analysis of Fragment2 was performed in 99 patients affected by TGCTs and in 100 cancer-free controls.

Alleles frequencies were determined by the gene counting method, as reported in Tables 3 and 4. All the alleles resulted in accordance with the Hardy–Weinberg equilibrium.

**Table 3:** Allele and genotype distribution of detected single nucleotide polymorphisms (SNPs) in the Fragment1

	Group T (*n* = 116)		Group C (*n* = 120)	
	%	No	%	No
C207T				
Allele				
C	1.00	232	1.00	240
T	0.00	0	0.00	0
Genotype				
CC	1.00	116	1.00	120
TT	0.00	0	0.00	0
G223A				
Allele				
G	0.728	169	0.638	153
A	0.272	63	0.363	87
Genotype				
GG	0.483	56	0.425	51
GA	0.491	57	0.425	51
AA	0.026	3	0,150	18
A288G				
Allele				
A	0.289	67	0.246	59
G	0.711	165	0.754	181
Genotype				
AA	0.259	30	0.167	20
AG	0.060	7	0.158	19
GG	0.681	79	0.675	81
T366C				
Allele				
T	1.000	232	1.000	240
C	0.000	0	0.000	0
Genotype				
TT	1.000	116	1.000	120
TC	0.000	0	0.000	0
CC	0.000	0	0.000	0

*Group T*, patients with testicular germ cell tumours; *Group C*, cancer-free controls

**Table 4:** Allele and genotype distribution of detected single nucleotide polymorphisms (SNPs) in the Fragment2

	Group T (*n* = 99)		Group C (*n* = 100)	
	%	No	%	No
C102A				
Allele				
C	0.631	125	0.650	130
A	0.369	73	0.350	70
Genotype				
CC	0.384	38	0.390	39
CA	0.495	49	0.520	52
AA	0.121	12	0.090	9
G172A				
Allele				
G	1.000	198	0.980	196
A	0.000	0	0.020	4
Genotype				
GG	1.000	99	0.960	96
GA	0.000	0	0.040	4
AA	0.000	0	0.000	0
C189T				
Allele				
C	0.631	125	0.650	130
T	0.369	73	0.350	70
Genotype				
CC	0.384	38	0.380	38
CT	0.495	49	0.540	54
TT	0.121	12	0.080	8
T245C				
Allele				
T	1.000	198	1.000	200
C	0.000	0	0.000	0
Genotype				
TT	1.000	99	1.000	100
TC	0.000	0	0.000	0
CC	0.000	0	0.000	0
C255A				
Allele				
C	1.000	198	1.000	200
A	0.000	0	0.000	0
Genotype				
CC	1.000	99	1.000	100
CA	0.000	0	0.000	0
AA	0.000	0	0.000	0
G371C				
Allele				
G	0.500	99	0.475	95
C	0.500	99	0.525	105
Genotype				
GG	0.242	24	0.200	20
GC	0.515	51	0.550	55
CC	0.242	24	0.250	25

*Group T*, patients with testicular germ cell tumours; *Group C*, cancer-free controls

Analysis of *PDE11A* SNPs revealed that all subjects (patients and controls) were homozygous for the SNPs C207T, T366C, G172A, T245C and C255A. Furthermore, the SNPs G223A and A288G appeared differently in cases and controls (χ^2^ *P* = 0.004 and *P* = 0.024, respectively). In contrast, the SNPs C102A, C189T and G371C showed a similar distribution in all subjects investigated.

**Fig. 1:**
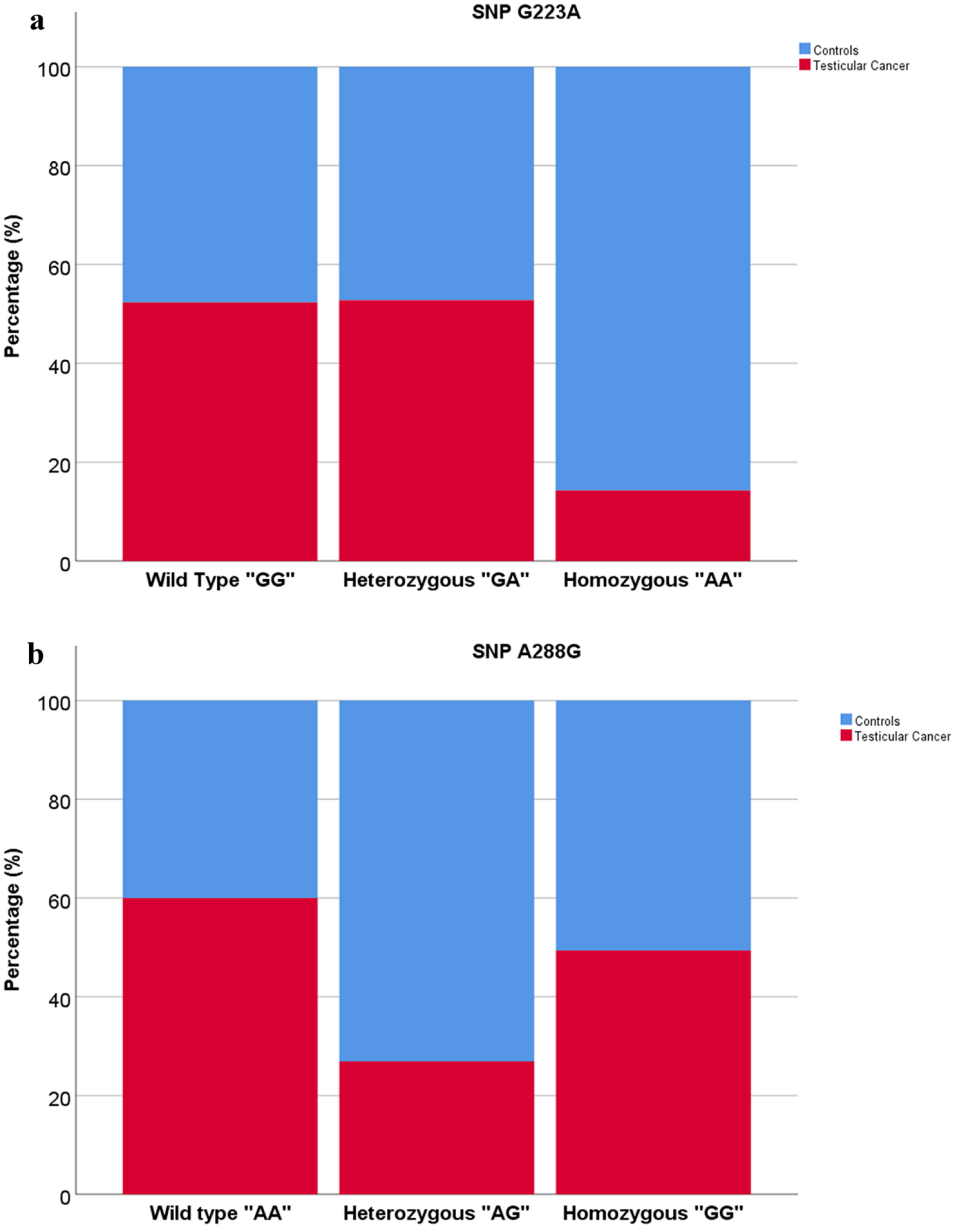
Distribution of SNP G223A and A288G in the two study groups: **a** distribution of SNP G223A between cases and controls, **b** distribution of SNP A288G between cases and controls

Then we used binary logistic regression models to analyze associations between testicular cancer and polymorphisms, considering the SNPs G223A, A288G, C102A, C189T and G371C as independent variables. This analysis revealed that only the SNP G223A in homozygosity and A288G in heterozygosity were significantly associated with a lower risk of testicular tumour (G223A homozygous: OR 0.123, 95% CI 0.034–0.451, *P* = 0.002; A288G heterozygous: OR 0.199, 95% CI 0.068–0.578, *P* = 0.003), as reported in Fig. 1a, b.

**Fig. 2:**
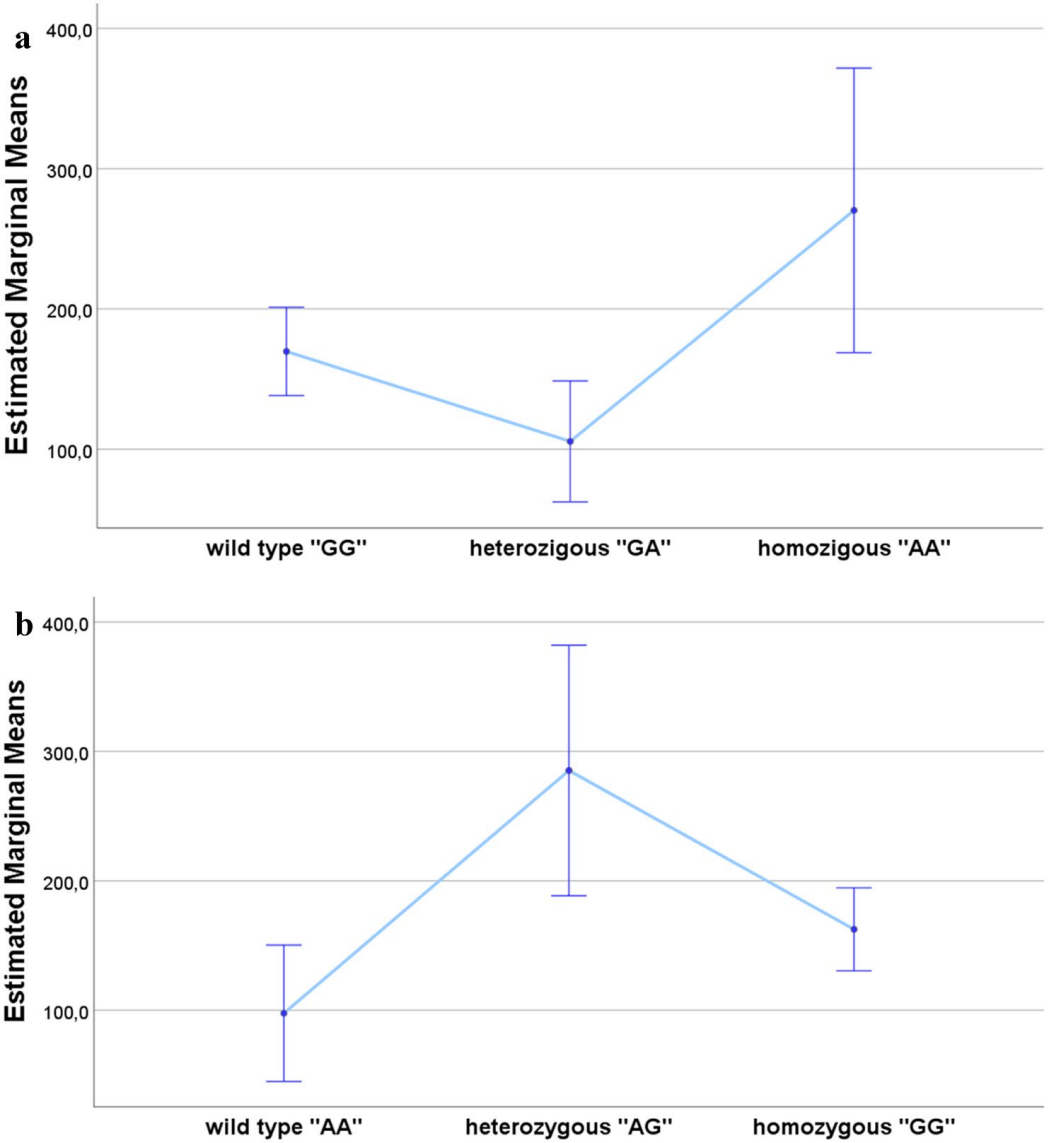
Association between total sperm number and the examined SNPs after correction for FSH values: **a** estimated marginal means ± standard errors of total sperm number (10^6^/ejaculate) for genotype G223A, **b** estimated marginal means ± standard errors of total sperm number (10^6^/ejaculate) for genotype A288G

Finally, using univariate models and after correction for FSH values, we identified an association between total sperm number and the SNPs G223A (Fig. 2a) and A288G (Fig. 2b) (*P* = 0.005 and *P* = 0.003, respectively; *R*^2^ = 0.241). In particular, total sperm number appeared higher in homozygote AA, in the case of G223A, and in heterozygote AG, in the case of A288G, than other genotypes.

## Discussion

TGCT is the most common malignancy in young adult males and its incidence has increased over the last 50 years. The risk for the development of these neoplasms is higher in Scandinavia, Switzerland and Germany, intermediate in the United States, Britain and Mexico, lower in Africa and Asia [23, 24]. However, the reasons for the differences in the incidence of TGCT among different ethnic groups are unknown [25].

Clinical and epidemiological studies suggest that several etiological factors could increase the susceptibility to testicular cancer, such as a family history of testicular cancer, exposure to environmental pollutants (endocrine disruptors) and cryptorchidism [10, 11, 26–30]. As the underlying molecular causes still remain unclear, we aimed to investigate in TGCT patients the genetic contribution of *PDE11A* polymorphisms, one of the putative genes behind of this complex and multifactorial disease. Moreover, we evaluated sperm parameters and hormone profile to assess testicular function in TGCT patients compared to cancer-free controls.

### Sperm parameters

Despite the mean sperm parameters of Group T are above the 5^th^ percentile of the WHO reference value, comparison with the healthy controls revealed a poorer semen quality in our cohort of testicular cancer patients. As TGCTs seem to arise from germ cell neoplasia in situ, which could originate from PGCs or gonocytes whose maturation is disturbed [2], testicular neoplasms may induce male infertility as a consequence of sperm parameters alterations.

The poorer semen quality observed in our TGCT patients is consistent with literature evidence, although the relationship between testicular neoplasms and infertility appears complex and controversial due to genetic, environmental and ethnic differences which could impact spermatogenesis. Over the last 20 years several studies evaluated sperm characteristics in these neoplasms before treatment, showing an impaired semen quality in TGCT patients [31–36].

It is noteworthy that, except for a few studies [37, 38], semen quality appears more compromised in testicular neoplasms than in other malignancies, even before beginning any antineoplastic treatment [32–34, 36]. This could be caused by TGCT itself through hormonal alterations and metabolic settings. In particular, *β*-human chorionic gonadotropin (*β*-hCG) could influence spermatogenesis directly or indirectly through hypothalamus-pituitary-gonad axis. It has been speculated that *β*-hCG could exert LH-like effects on Leydig cells and could induce a feedback on hypothalamus-pituitary axis impairing gonadal function [32, 39]. As reported in the literature, the presence of a compromised spermatogenesis in TGCT patients with higher serum *β*-hCG levels would confirm this hypothesis [32, 39]. Malignancy might also result in malnutrition, with consequent psychological complications and deficiencies in vitamins and minerals needed for a proper testicular function. Finally, spermatogenesis might be negatively influenced by periods of fever and by tumour release of cytokines. All these factors expose testicular cancer patients at the highest risk of having reduced semen quality before treatment, which can further negatively impact fertility making sperm cryopreservation an important clinical option for male fertility preservation [32, 35, 36, 40–53].

### Hormone profile

The presence of a tumour is supposedly associated with an altered hormone profile due to different causes, such as a dysregulated hormonal secretion or a release of hormones by the tumour itself.

In support of this hypothesis, patients affected by TGCTs are more likely to show higher levels of FSH and LH and lower levels of testosterone, an endocrine pattern which also characterizes infertile men [54]. It should be stressed that orchiectomy, testicular dysgenesis syndrome, treatment after orchiectomy and aging could play a key role in the increase of prevalence of hypogonadism in these patients.

The hormone profile appeared significantly altered in Group T in comparison with the cancer-free controls: in particular, testicular cancer patients showed higher serum gonadotropins levels with reduced testosterone, confirming previous literature observations [55, 56].

Although the hormone profile was altered, our cohort of testicular cancer patients showed mean sperm parameters lower than the controls but within the reference limits. As also demonstrated in animal models, this evidence can be explained through a compensatory mechanism: orchiectomy may cause a rapid decline in inhibin B levels due to the halving of the number of Sertoli cells; this provides the stimulus for a surge in FSH secretion by the pituitary which may induce proliferation of germ cells in contralateral testis and an increase of testicular volume, under physiological functional conditions [57–59]. For this reason, despite the known association between BMI and hypogonadotropic hypogonadism, we found that in this cohort of TGCT patients BMI is positively associated with gonadotropins.

### *PDE11A* analysis

The frequent diagnosis in young men with a positive family history for TGCTs and the increased risk for the children and siblings of men with testicular cancer point to a genetic basis of these neoplasms [26–28].

Linkage analyses suggest that the susceptibility may result from the interaction of multiple common and low-penetrance genetic variants [60–62] and one of the main candidate genes is *PDE11A*, expressed in testicular tissue in all four known isoforms [15].

Studies of adrenal, prostate and testicular cancer have suggested that *PDE11A* variants may represent susceptibility modifiers rather than direct and sufficient causes of these neoplasms [63]. This gene may play a key role also in spermatogenesis and fertilization potential, as suggested by observation that *Pde11a* knockout mice displayed reduced sperm concentration, rate of forward progression, percentage of live spermatozoa and increased premature/spontaneous capacitance [17]. These evidences suggest a role for *PDE11* in testicular tissue.

Inactivating *PDE11A* variants induce alterations in cAMP pathway increasing the levels of this cyclic nucleotide, which may promote TGCT development similarly to what has been observed in non-germ cell-derived testicular tumours, such as in Leydig cell hyperplasia, McCune-Albright syndrome and Carney complex-associated Sertoli cell tumours [20, 21].

The role of *PDE11A* polymorphisms has been explored in various diseases but recent studies highlighted their contribution also in testicular cancer. In 2009, Horvath et al*.* analyzed the *PDE11A* coding sequence in 95 patients with familial and bilateral TGCT, finding a significantly higher frequency of the non-synonymous substitution p.V820M among testicular cancer patients than control subjects [12].

Subsequently, Pathak et al*.* sequenced the *PDE11A* coding region in 259 patients with both familial and sporadic TGCT, detecting 55 variants including p.V820M and p.K568R, which were present only in cases and not in controls [14]. It is noteworthy that *PDE11A* variants identified in these studies resulted in reduced PDE activity and increased cAMP levels modifying the TGCT risk not only in familial and bilateral form, but also in sporadic form.

In our caseload, we aimed to identify p.V820M and p.K568R, two polymorphisms detected in the aforementioned studies, to confirm their role in patients affected by unilateral and bilateral sporadic TGCTs. Both SNPs affect critical sites of the enzyme: in particular, p.V820M (Fragment1) is placed in the catalytic domain, while p.K568R (Fragment2) in GAF-B domain required for enzyme oligomerization.

None of our TGCT patients and controls showed the two SNPs investigated. However, *PDE11A* sequencing revealed ten new polymorphisms not yet associated with testicular cancer before: four for the Fragment1 (C207T, G223A, A288G, T366C) and six for the Fragment2 (C102A, G172A, C189T, T245C, C255A, G371C).

The discrepancies in the genetic results between our study and literature could arise from differences in the alleles frequencies due to geographical distribution. Although the populations in question have Caucasian origin, environmental factors and genetic recombination may have diversified the genetic profiles over time. Furthermore, it should be stressed that we analyzed almost exclusively patients affected by unilateral sporadic TGCTs, whereas the studies reported in the literature focused mainly on bilateral familial cases.

As most of the new SNPs detected in our study are uniformly present in the caseload as a whole, it is plausible that they are constitutive polymorphisms. The only two SNPs showing a different significant distribution between case and controls are G223A and A288G, both localized in the Fragment1 such as p.V820M detected in the aforesaid studies. In particular, A288G is an intronic variant, while G223A is not present in dbSNP database. Therefore, it was not possible to identify it as an intronic or exonic variant. Examining putative associations between these two SNPs and pathological conditions, we found that only A288G has previously been related to antidepressant treatment response [64].

Analysis of associations between testicular cancer and *PDE11A* polymorphisms revealed that the homozygote AA, in the case of G223A, and the heterozygote AG, in the case of A288G, were significantly associated with a lower risk of testicular tumour than the other genotypes. Moreover, they displayed a significant positive correlation with total sperm number. As these two genotypes resulted associated with a lower risk of TGCTs, we suggest that they could improve PDE11A function in the presence of risk factors for testicular cancer development, such as cryptorchidism, endocrine disruptors, etc. Hence, this function would be opposite to that induced by the SNPs detected by Horvath et al*.* 2009 [12] and Pathak et al*.* 2015 [14], which reduce enzymatic activity increasing cAMP levels and TGCT risk. The putative protective role of these two genotypes can be deduced from the finding of reduced PDE activity and consequent increased cAMP levels which also characterize other tumour settings [20, 21].

Moreover, the association with total sperm number allows us to hypothesize that these genetic variants could influence, not only the onset of testicular neoplasms, but also the spermatogenesis process.

However, as the underlying molecular mechanisms are still unclear, it is plausible to assume that additional factors involved in cAMP signaling could play a pivotal role. An example is provided by CREM (cAMP-response-element modulator), a transcription factor responsive to the cAMP signal transduction pathway which represents a master regulator of key testis-specific genes necessary for spermatogenesis [65, 66].

## Conclusions

TGCTs are complex neoplasms whose aetiology is multifactorial. In our study, we observed that TGCT patients showed an altered hormone profile and a poor semen quality, although the sperm parameters were within the reference limits. Moreover, we identified ten new *PDE11A* polymorphisms, two of which significantly associated with a lower risk of testicular tumour. This result remarks that the genetic contribution could be critical in the susceptibility to these neoplasms.

Nonetheless, PDE11A role in testis is still unclear and the contribution of additional factors involved in cAMP signaling not investigated in our study cannot be excluded. Hence, further investigations are needed to elucidate the underlying molecular mechanisms and to clarify how alterations in cAMP pathway could influence the TGCT risk.
